# Electrophysiological Characteristics of a SCN5A Voltage Sensors Mutation R1629Q Associated With Brugada Syndrome

**DOI:** 10.1371/journal.pone.0078382

**Published:** 2013-10-22

**Authors:** Zhipeng Zeng, Jieqiong Zhou, Yuxi Hou, Xiaojing Liang, Ziguan Zhang, Xuejing Xu, Qiang Xie, Weihua Li, Zhengrong Huang

**Affiliations:** Department of Cardiology, the First Affiliated Hospital of Xiamen University, Xiamen, China; Xuzhou Medical college, China

## Abstract

Brugada syndrome (BrS) is an inherited arrhythmogenic syndrome leading to sudden cardiac death, partially associated with autosomal dominant mutations in *SCN5A*, which encodes the cardiac sodium channel alpha-subunit (Na_v_1.5). To date some *SCN5A* mutations related with BrS have been identified in voltage sensor of Na_v_1.5. Here, we describe a dominant missense mutation (R1629Q) localized in the fourth segment of domain IV region (DIV-S4) in a Chinese Han family. The mutation was identified by direct sequencing of *SCN5A* from the proband’s DNA. Co-expression of Wild-type (WT) or R1629Q Na_v_1.5 channel and hβ1 subunit were achieved in human embryonic kidney cells by transient transfection. Sodium currents were recorded using whole cell patch-clamp protocols. No significant changes between WT and R1629Q currents were observed in current density or steady-state activation. However, hyperpolarized shift of steady–state inactivation curve was identified in cells expressing R1629Q channel (WT: V_1/2_ = -81.1 ± 1.3 mV, n = 13; R1629Q: V_1/2_ = -101.7 ± 1.2 mV, n = 18). Moreover, R1629Q channel showed enhanced intermediate inactivation and prolonged recovery time from inactivation. In summary, this study reveals that R1629Q mutation causes a distinct loss-of-function of the channel due to alter its electrophysiological characteristics, and facilitates our understanding of biophysical mechanisms of BrS.

## Introduction

Brugada syndrome (BrS) is a heritable arrhythmia syndrome characterized by an ST segment elevation in ECG leads V_1_ to V_3_ and an increased risk for sudden cardiac death (SCD) due to ventricular fibrillation (VF) [1].BrS is estimated to account for 4% of all SCD and 20% of unexplained sudden deaths without obvious structural heart disease [2].To date, more than ten different genes have been associated with BrS [3,4].The major disease gene for BrS is *SCN5A*, a gene encoding the primary alpha-subunit of the cardiac sodium channel (Na_v_1.5), accounting for 10-30% of subjects with BrS carrying a *SCN5A* gene mutation with autosomal dominant inheritance [2,5]. Recently, over 300 *SCN5A* mutations have been identified in BrS patients [6-8]. The BrS mutant channels which have been characterized so far in vitro revealed loss-of-function by a variety of mechanisms, including reduced current density or represented abnormal biophysical characteristics [7-10]. However, despite many studies, the molecular and cellular mechanisms underlying BrS are still not completely known.

Voltage-gated sodium channels play a key role in initiation and propagation of cardiac action potential that is essential for the rhythm beating of the heart. Moreover, mutations in *SCN5A* and auxiliary subunits genes (*SCN1B*-*4B*) have been found to be associated with a various inherited arrhythmia syndromes that includes BrS, long QT syndrome type 3 (LQT3), progressive cardiac conduction defect (PCCD), sick sinus node syndrome, atrial fibrillation and even dilated cardiomyopathy (DCM) [10]. The identification of *SCN5A* mutation in patients with inherited arrhythmogenic syndromes is critical for the understanding of the pathogenesis of arrhythmias. It could provide practical information that is very helpful for optimal patient management and risk stratification [11,12]. In addition, understanding the structural-functional relationship of the Na_v_1.5 will shed new light on exploiting new therapeutic drugs for *SCN5A* channelopathies.

In this study，we described a Chinese Han family with two male patients diagnosed of BrS, one of which died of SCD. Screening of the *SCN5A* gene from the proband resulted in the detection of a heterozygous mutation R1629Q in the voltage sensor of domain IV. To understand the molecular mechanisms determining the malignant phenotype, we analyzed biophysical properties of mutant sodium channel in HEK293 cells.

## Methods

### Ethics Statement

This study was approved by the Medical Ethical Committee of the first affiliated hospital of Xiamen University (Xiamen, China) and conformed with the principles outlined in the Declaration of Helsinki. Blood samples were obtained after written informed consent.

### Clinical Data

**Figure 1:**
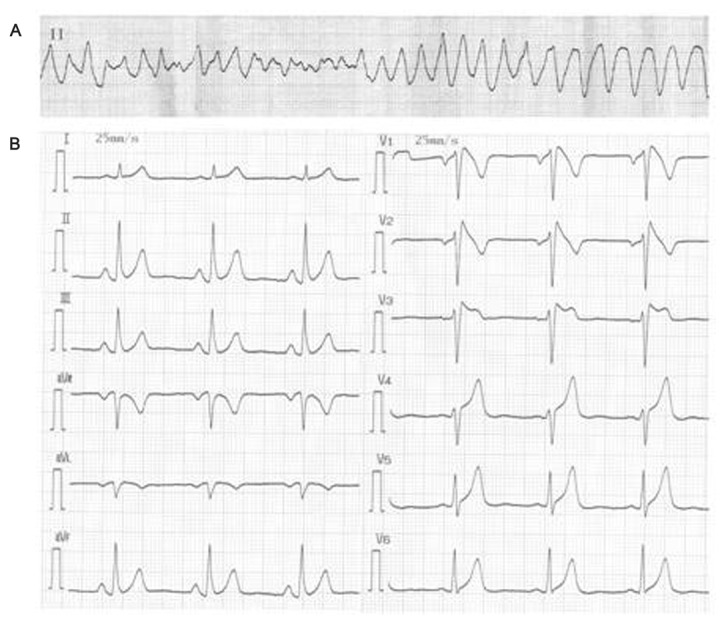
Twelve-lead ECG recording of the proband with Brugada syndrome. (A): ECG monitor strip of the proband showing polymorphic ventricular tachycardia (254 bpm) recorded at the arrival to the emergency room (B): Twelve-lead ECG recording of the proband with Brugada syndrome at baseline, showing prominent coved ST-segment elevation, following a negative T wave in V_1_-V_2_ leads ,and ST-segment saddleback elevation in V_3_ lead (Type I BrS ECG).

A family composed of 9 subjects that belong to the Chinese Han population (six males, three females, mean age 47.2 ± 25.5 years) underwent physical examination, basal bio-chemical marker detection, resting 12-lead ECG, 24h Holter ECG, echocardiogram, and genetic screening for the *SCN5A* mutation. The family was studied after the investigation of the proband (55-year-old man) was admitted to the hospital due to the onset of a sustained polymorphic ventricular tachycardia (PVT) and in whom baseline ECG showed ST segment coved elevation in V_1_-V_2_ (Type I) and incomplete right bundle branch block (Figure 1). The BrS patients underwent an exercise stress test, invasive cardiac evaluation with right ventricular angiography, electrophysiological study, MRI and hemanalysis showed no evidences of structural heart disease. 

### Mutation Analysis of SCN5A in BrS

 Genomic DNA was extracted from blood sample using Puregene DNA purification Kit (Tiangen biotech, Beijing, China). Previously published primer pairs were used to amplify all exons and exon-intron boundaries of *SCN5A* gene from genomic DNA [13]. Polymerase chain reaction (PCR) products were purified (Tiangen biotech, Beijing, China) and they were directly sequenced for mutation using ABI Prism 3730XL DNA sequencer (Applied Biosystems, Foster City, CA, USA).The DNA sequence and amino acid were based on the *SCN5A* transcript NM_198056.2 [14]. DNA samples from 150 healthy Chinese Han individuals (300 alleles) were used as control samples.

### Mutagenesis and Heterologous Expression

Wild-type (WT) human heart *SCN5A* cDNA (Uniprot reference: Q14524-1) and Na_v_1.5 channel hβ_1_-subunit SCN1B cDNA (Uniprot reference: Q07699-1) subcloned into pcDNA3 and pIRES2-DsReD vector for mammalian expression, respectively. Both the plasmids, generous gifts from Dr. Qing K. Wang, were described previously [15,16]. R1629Q mutation was created by site-directed mutagenesis using overlap extension PCR on the basis of pcDNA3-hH1 template and the following primers (mutation underlined): 

5’-GGCCTATTTGGGCCAGGCGGATGAC-3’and 

5’-CGCCTGGCCCAAATAGGCCGCATCCT-3’ 

The mutant clone was confirmed by direct sequencing. For patch-clamp studies, HEK293 cells, a kind gift from Dr. Qing K. Wang, were cultured as previous methods [16]. Cells were transiently cotransfected with 0.8 µg pcDNA3-*SCN5A* (WT or mutants) and 0.8 µg of pIRES2-DsReD-SCN1B as a reporter gene using Lipofectamine 2000 (Invitrogen, Carlsbad, CA, USA), following the manufacturer’s instructions. All experiments were performed 48 hours after transfection. 

### Cellular Electrophysiology

Sodium currents were recorded at room temperature (22°C-25°C) using whole cell patch-clamp technique as previous methods [16]. The experiments were conducted with an MultiClamp™ 700B amplifier and pClamp 10.1/Digidata 1440A acquisition system (Molecular Devices, Sunnyvale, CA, USA). The data was analyzed by OriginPro8 software (OriginLab Corporation, Northampton, MA, USA). The curves of activation and steady-state inactivation were fitted to a Boltzmann function, y=1/{1+exp[(*V*_m_-V_1/2_)/k]},where y is the normalized current or conductance, *V*_m_ is the membrane potential, *V*_1/2_ is the voltage at which half of the channels are activated or inactivated and k is the slope factor [7,16,17]. Recovery curves from inactivation were fitted with two-exponential equation, *I*(t)/*I*_max_=A_1_×exp(–t/τ1)+A_2_×exp(–t/τ2), in which values for A and τ refer to amplitudes and time constants, respectively [16,17]. Decay characteristic of the fast transient current was fitted to a single-exponential equation over a range of voltages from -50 mV to -30 mV, from where time course were obtained [17].

### Statistical Analysis

Statistical data were reported as means ± standard error (SE) unless otherwise noted. Statistical comparisons between wild-type and mutation groups were tested using the unpaired Student’s t-test. *P*< 0.05 was considered statistically significant.

## Results

### Clinical Evaluation

**Figure 2:**
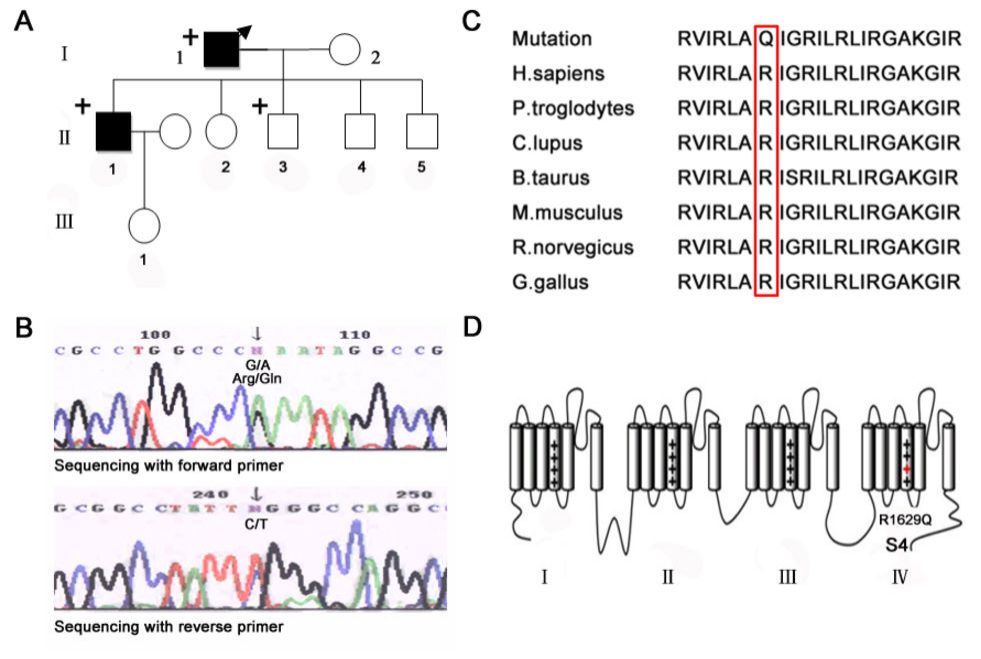
Family pedigree and R1629 is highly conserved among mammal species, localized in the voltage sensor of domain IV. (A) Pedigree of the Chinese Han family. Male is shown as squares, female shown as circles. Filled symbols marked individual infected by BrS. Symbol”+” marked variation carrier for *SCN5A* *c*.4886G>A. The proband was male member with BrS, shown with an arrow (B) Chromatograms demonstrating heterologous peaks in nucleotide sequence. A heterozygous nucleic acid substitution (*c*.4886G>A) was screened in exon28 of the *SCN5A* gene from the proband’s DNA sample (C) Sequence alignment of the DIV-S4 around the altered amino acid residue 1629 showed a high degree of conservation when compared with orthologs of *SCN5A* among mammal species (D) Location of R1629Q mutation in the predicted topologic structure of the Na_v_1.5 channel, marked by a red “+” sign.

The proband (I1, Figure 2A), a 56-year-old man, was admitted to hospital because of PVT (Figure 1A) terminated with electrical cardioversion. At rest, electrocardiogram showed sinus rhythm and a prominent coved ST-segment elevation >2mm, following a negative T wave in V_1_-V_2_ leads, in accordance with BrS type I ECG (Figure 1B). The proband’s son (II1) showed Type I pattern characterized by ST-segment elevation in right precordial leads and had a history of unexplained syncope occurring at rest. Despite carrying R1629Q mutation, individual II3 was asymptomatic and just showed suspicious Type II ECG pattern. In addition, the remaining family members had normal baseline ECG, X-ray and 2D-echocardiogram, screening no mutation in *SCN5A* gene. The proband refused the therapy of the implantable cardioverter defibrillator due to financial condition. During the follow-up (22 months) the patient died of SCD.

### Molecular genetics

Mutation screening was performed on genomic DNA in the exons and exon-intron boundaries coding sequences of *SCN5A* gene by direct sequencing. A single heterozygous nucleotide transition (G-to-A) at position 4886 of the *SCN5A* gene (*c*.4886 G>A) was identified in proband’s DNA. This base transition results in arginine replaced by glutamine at position 1629 (*p*.R1629Q) of the Na_v_1.5 channel in the affected cases (Figure 2B).Interestingly, the R1629Q mutation localizes on the fourth trans-membrane segment of domain IV (DIV-S4), regarded as voltage sensor of sodium channels. R1629 is highly conserved within in Na_v_1.5 channel of other mammal species by sequence alignment (Figure 2C). Absence of *SCN5A c*.4886A allele in 150 control individuals (300 chromosomes), Human Gene Mutation Data base (HGMD) [18], Ensembl [19] and HapMap [20] suggested that it was a possible mutation causing BrS rather than a rare polymorphism. The mutation was also confirmed in the proband’s two sons (II1; II3).

### Results of the Cellular Electrophysiological Study

**Figure 3:**
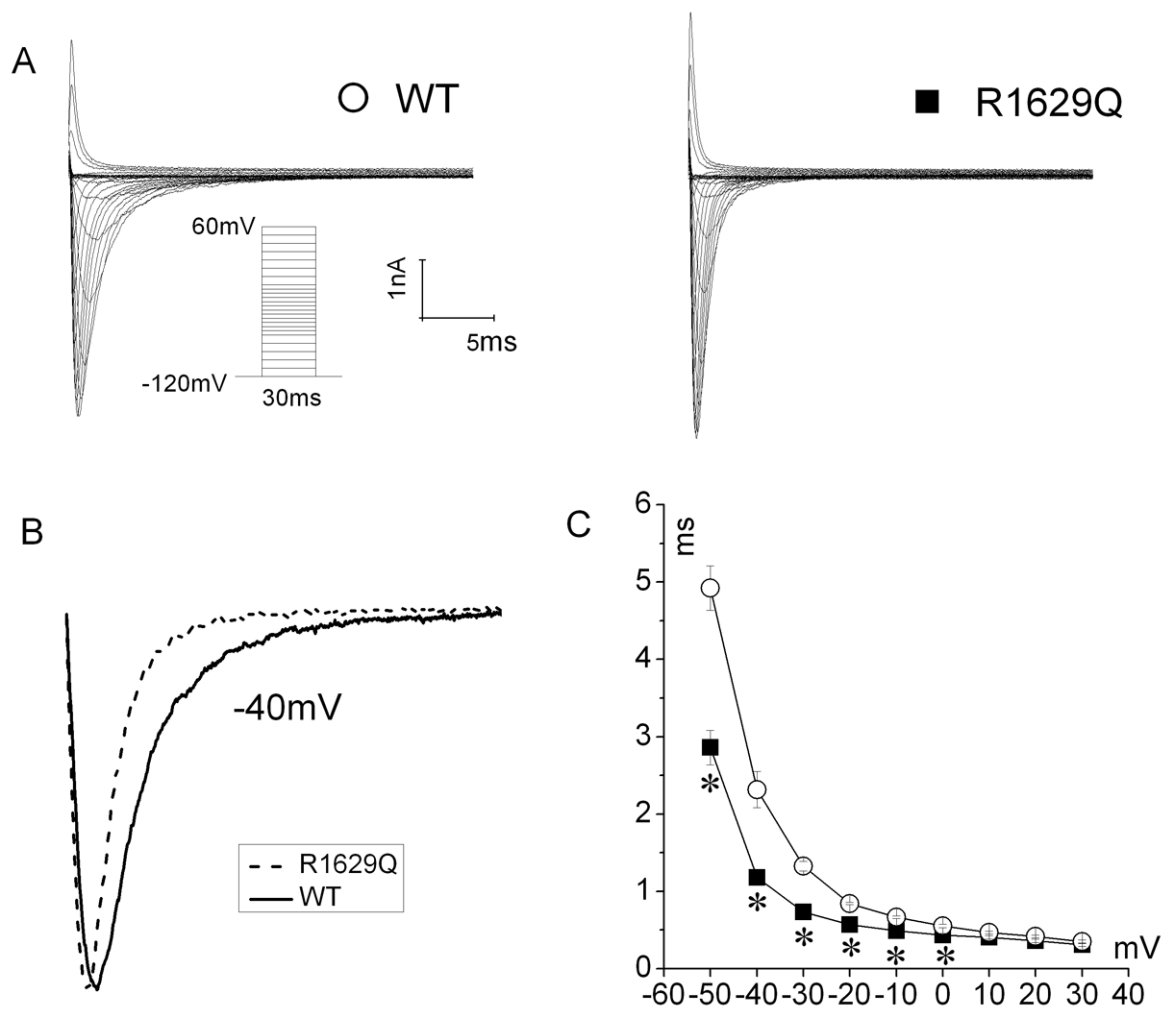
R1629Q mutation affects the time course of current decay. (A) Example current traces of WT (open circle) and R1629Q (filled square) channels. (B) A shows typical normalized current traces recorded in response to - 40mV step for WT (solid line) and R1629Q (dash line) SCNA5 channels. The decay of I_Na_ was fit to a mono-exponential (τ time constants) decay function. Note that the decay of the R1629Q channels was faster than WT. (C) Current decay time constants for WT (open circle) and R1629Q (filled square) between -50mV and 30mV.*P<0.05 vs. WT in this study. Results are expressed as means±SE for 6 to 11 cells.

We subsequently compared the biophysical properties of wild-type recombinant human Na_v_1.5 channels (WT) or R1629Q channels in the presence of hβ1 subunit in HEK293 cells at room temperature (22°C-25°C). To control membrane size, sodium currents were recorded in the whole-cell patch clamp configuration and current amplitudes were divided by the membrane area calculated from the electrode tip size. Both WT and R1629Q channels give rise to typical inward currents that completely deactivate within milliseconds (Figure 3A). In fact, we observed no significant difference in current density between WT and R1629Q (Table 1).Therefore, the R1629Q mutation is probable not a gross structural mutation disrupting channel trafficking and/or the stability of the channel.

**Table 1:** The Kinetic Parameters for WT and R1629Q channels.

	I_Na_ at -20mV		Activation			Inactivation			Decay(-40mV)		Recovery from inactivation		
	*pA/pF*	*n*	V_1/2_(mV)	*k*	*n*	V_1/2_(mV)	*k*	*n*	τ (ms)	*n*	τf(ms)	τs(ms)	*n*
WT	-336.7±26.9	30	34.5±1.5	6.0±0.3	8	-81.1±1.3	5.2±0.1	13	2.32±0.24	6	3.2±0.2	104.1±20.0	11
R1629Q	-298.7±27.0	22	32.2±1.4	6.4±0.2	11	-101.7±1.2**	5.8±0.1*	18	1.18±0.1*	11	22.5±1.0**	139.0±14.0	12

Each entry is the mean ± SE obtained from n experiments. For the Boltzmanm fit (activation and inactivation) the parameters are V_1/2_ midpoint and slope factor (k). For the double-exponential fit (recovery) the parameters are: τf the fast time constant; τs the slow time constant; for the single exponential fit (decay) the parameter is: τ the time constant. All parameters were analyzed by t-test. * *P*<0.05 vs WT, ** *P*<0.01 vs WT.

**Figure 4:**
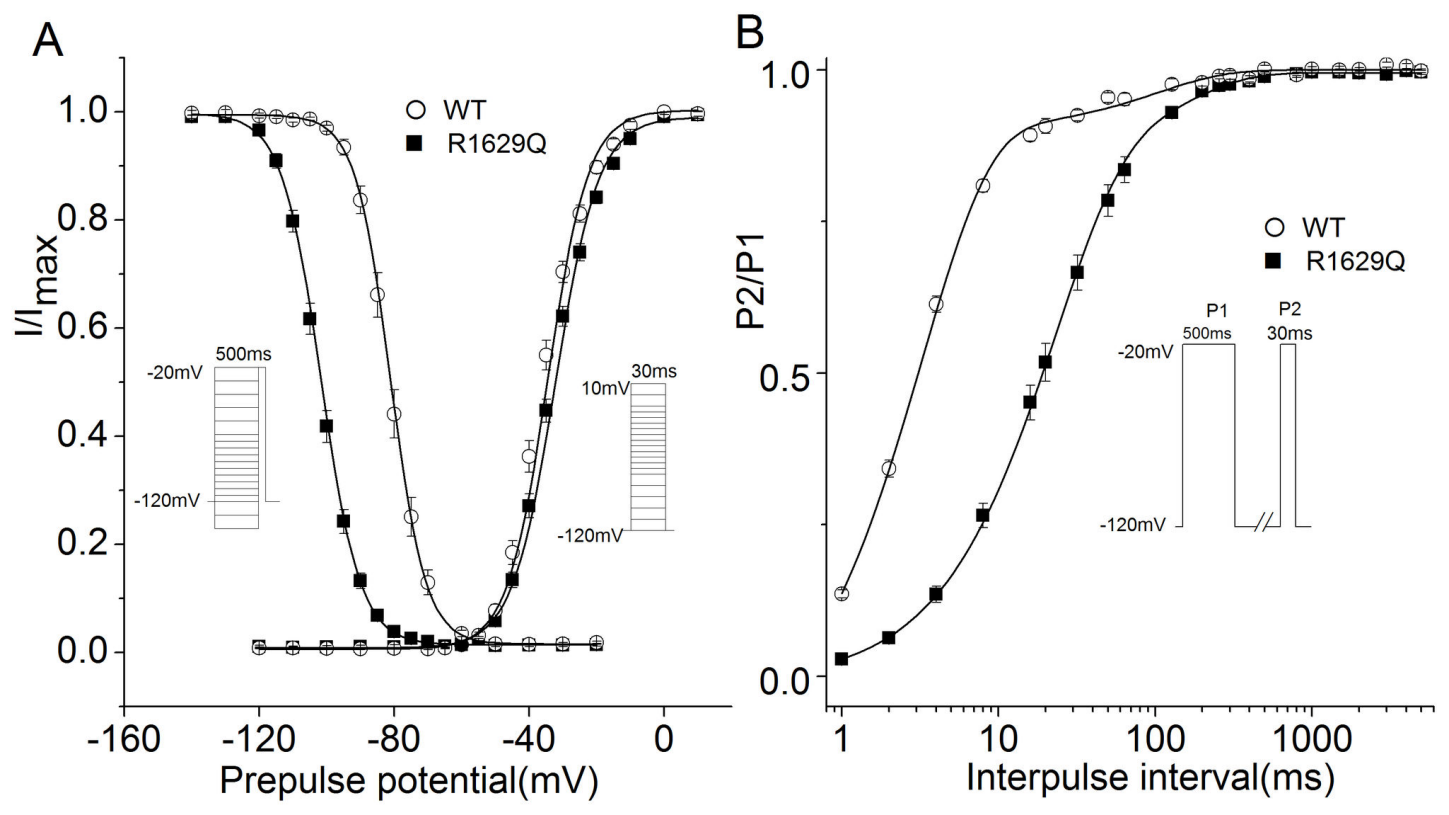
R1629Q alters Na_v_1.5 channel inactivation kinetics and prolongs the recovery time from inactivation. (A) Voltage dependence of steady-state activation and inactivation curve of WT (open circle) and R1629Q (filled square) channels. The protocol was shown in the Inset and Values for the half-maximal voltage (V_1/2_) were provided in the table 1. (B) Time courses of recovery from inactivation were investigated using the two-pulse protocol show in the inset. Time constants are as follows: WT, τf=3.2±0.2ms, τs=104.1±20.0ms,n=11;R1629Q,τf=22.5±1.0ms,τs=139.0±14.0ms,n=12.(Comparison of τf between groups is statistically significant. *P*<0.01).Results are presented as mean±SE.

Despite no change between WT and R1629Q channel is observed in current density, the R1629Q mutation represents abnormal kinetic properties of Na_v_1.5 channel (Table 1). Accordingly, R1629Q has no significant effect on the channel activation, as can be seen from the voltage dependence activation (Table 1, Figure 4A). However, Na_v_1.5 R1629Q displays a 20.6 mV negative shift of the steady-state inactivation curve toward more negative potentials (WT: V_1/2_ = -81.1 ± 1.3 mV, n = 13; R1629Q: V_1/2_ = -101.7 ± 1.2 mV, n = 18; *P*<0.01), and a faster onset of inactivation measured as time course of current decay compared with WT Na_v_1.5 (Table 1, Figure 3), that reveals that Na_v_1.5 R1629Q induces a significant preferential transition into the deactivation. We compared the differences in the kinetics of recovery from inactivation induced by a 500-ms depolarization to -20 mV, followed by a variable recovery interval at -120 mV for WT or R1629Q channel. The recovery curve for both channels is most in keeping with two exponential components where the fast time constant τf and the slow time constant τs are acquired. The τf in R1629Q channel is significantly slower as compared to WT channel (Table 1, Figure 4B).

**Figure 5:**
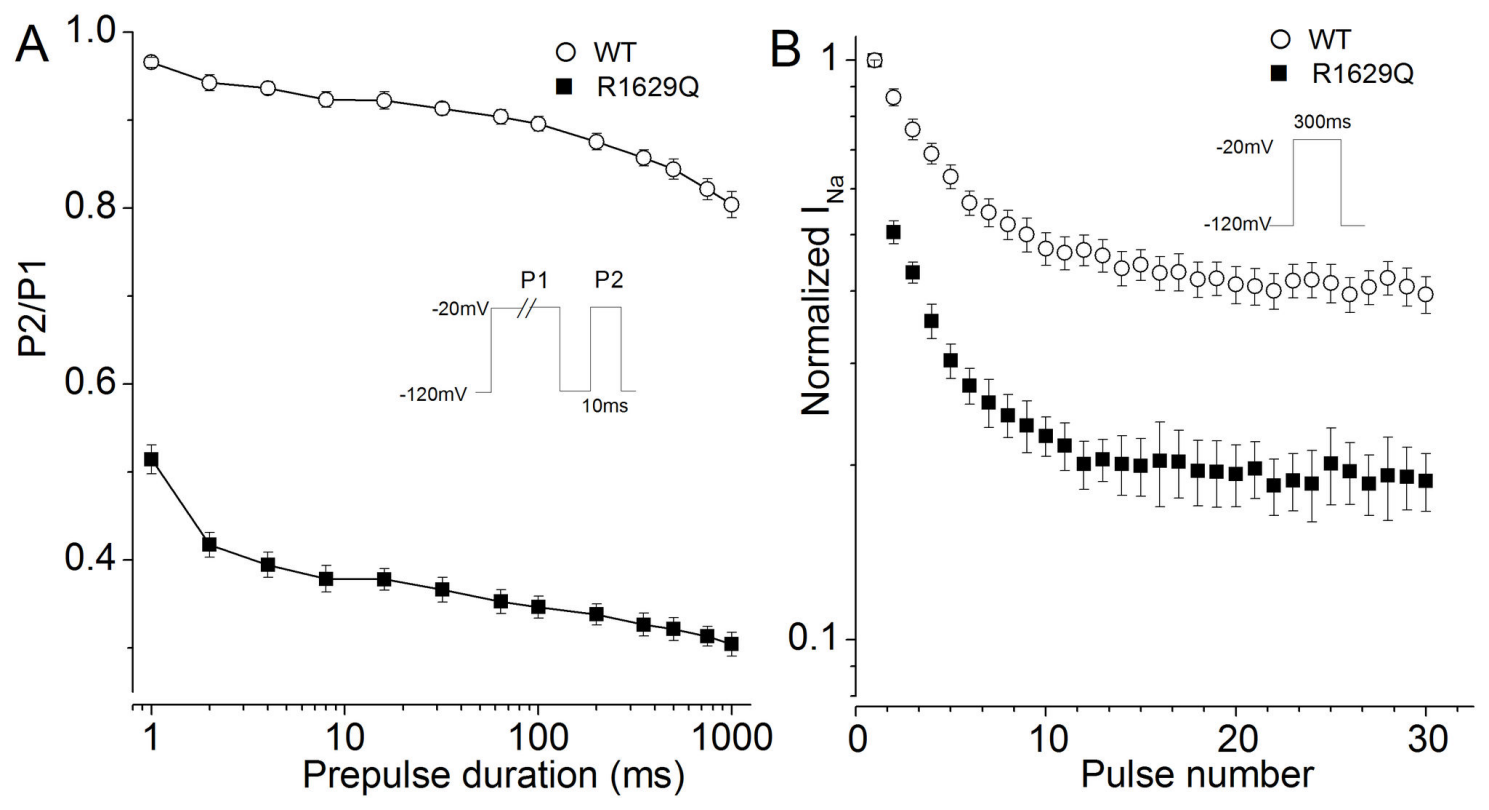
R1629Q enhances Na_v_1.5 channel into intermediate inactivation. (A) Time dependence of intermediate inactivation for WT and R1629Q.The two-pulse protocol is shown in the Inset. Differences between WT (open circle, n=9) and R1629Q (filled squre, n=8) were significant at all prepulse durations from 1 to 1000ms. (B)Activity-dependent loss of sodium channel availability. Sodium currents are recorded during series trains of 300ms depolarization to -20mV from holding potential -120mV.Currents are normalized to the value obtained after the first pulse and plotted against the successive pulse number. The residual normalized current level recorded after 30th pulse is 39.4%±2.1% for WT (open circles, n=14) and 18.7%±2.1% for R1629Q (filled squares, n=15) (*P*<0.01).

In addition, we also examined whether R1629Q channel exhibits an alteration in intermediate inactivation which might be a particular biophysical mechanism of BrS. Intermediate inactivation process, defined as a form of inactivation occurring over a time period of a few hundred milliseconds to tens of seconds, has an important physiological implications in determining the channels availability for firing action potential [21]. Some studies described that certain *SCN5A* mutations (T1620M, 1795insD et al) associated with BrS functionally reduced cardiac sodium current in the myocardium by enhancing the entry process of the channel into an intermediate inactivated state [21,22]. In addition, some investigators revealed that S4 segment of the domain IV was responsible for fast and slow inactivation of sodium channels [23-26].To find whether R1629Q mutation affects the process, we recorded the time dependence of the intermediate inactivation state for the WT or R1629Q channel. With the prolongation of the pulse duration, sodium currents decreased much more notably in cells expressing R1629Q than in those transfected with WT. Differences between WT and R1629Q were significant at all prepulse durations at the level of *P*<0.05. After a 1000-ms depolarization to -20 mV then a 10-ms repolarizing step to -120 mV to allow recovery from fast inactivation, the proportional decrement in channel availability was 3.6-fold greater for R1629Q than WT (WT 19.6+1.5%, n=9; versus R1629Q 69.6+1.4%, n=8, *P*<0.01 Figure 5A), indicated the R1629Q mutant showed a significant preferential transition into the intermediate inactivation state than WT. As shown in Figure 5B, to elucidate the potential pathophysiological significance of this effect, we recorded peak sodium currents during a series of 300–ms test depolarization to -20 mV at a rate simulating a cardiac cycle length of 0.32 seconds. R1629Q mutant channels show a significantly greater degree of activity-dependent loss of availability. The residual normalized current levels recorded after 30th pulse are 39.4%±2.1% for WT (open circles, n=14) and 18.7%±2.1% for R1629Q (filled squares, n=15) (*P*<0.01). Therefore, our results exhibit that the mutation R1629Q causes Na_v_1.5 channels enhanced accumulation of I_Na_ into an intermediate inactivated state. To sum up, R1629Q mutation may alter the electrophysiological properties of Na_v_1.5 channels, causing the loss-of-function of sodium channels. 

## Discussion

In this study, we identified a *SCN5A* mutation (R1629Q) associated with a severe clinical cardiac disorder BrS in a Chinese Han family. Although the R1629Q mutation was recently identified in a sporadic BrS patient, convincing pathophysiological mechanism attributed to BrS has still not been investigated [6]. The R1629Q mutation localizes in a highly conserved position which consists of an arginine to glutamine substitution for the third basic residue in DIV-S4, counting from the extracellular (amino) end of S4. Currently, there are some mutations associated with *SCN5A* channelopathies localized in this voltage sensor domain [6,10]. We analyze biophysical properties of mutated R1629Q Na_v_1.5 channel in vitro. Our data reveals that the mutation R1629Q induces an evident loss-of-function of cardiac sodium channel by shifting voltage dependence of inactivation, enhanced intermediate inactivation as well as prolonged recovery time from inactivation. 

In the family, the two affected individuals (I1 and II1) were heterozygous carriers of the *c*.4886A allele. The mutation carrier (II3) has not been diagnosed as BrS due to merely showing saddleback (Type II) ECG pattern. However, there were several findings that supported the pathogenic nature of this heterozygous mutation: a) only individuals heterozygous for the missense mutation showed some characteristics of BrS; b)*SCN5A c*.4886G>A was not screened in samples from a large control group and some DNA databases, therefore eliminating a single nucleotide polymorphism; c)the R1629 residue in the voltage sensor segment4 of domain IV was highly conserved across mammal species (Figure 2C); d)the same mutation in *SCN5A* gene was recently identified in a sporadic patient with BrS; and e) significant biophysical defect in the mutant sodium channel protein also demonstrated the notion that *SCN5A* R1629Q was indeed a pathogenic mutation. The heterozygous mutation carrier II3 showed normal cardiac function without syncope and cardiac arrhythmia, and rejected to perform ajmaline challenge test. Due to the variability of ECG pattern in BrS patients and no performing pharmacologcal test in the Ⅱ3 individual, the man cannot rule out the diagnosis of BrS [27].

Abnormalities of right ventricular for outflow wall motion and contraction have been detected in some patients with BrS [28-30]. However, to date most people still consider BrS is non-organic and heterogeneous heart rhythm disorder. The most attractive and well-convinced hypothesis to explain the basic electrophysiological mechanism of BrS involves loss-of-function of cardiac sodium channel, resulting in the imbalance of inward and outward current especially by the existence of a transmural repolarization gradient in the right ventricular wall where disproportionate expression of the transient outward current [21,31]. More than 300 *SCN5A* mutations are identified in BrS [6-8]. Most of them are missense mutations (193 mutations, account for 66%), and tend to cluster around the trans-membrane spanning region [6].These studies revealed that *SCN5A* mutations linked to BrS resulted in the loss-of-function of the Na_v_1.5 channels in heterologously expressed systems [7-10].Various distinct mechanisms are known to produce loss-of-function, including reduced expression of the channel in the plasma membrane to decrease sodium current, shifted curves of the voltage dependence of the channel activation or inactivation, or changed the characterizes of channel kinetic [10]. 

In the presence of the hβ1 subunit, the current density of the R1629Q channel is similar to WT. However, the mutation R1629Q leads to a distinct loss-of-function of Na_v_1.5 channels by distinct abnormal biophysical characteristics, such as hyperpolarizing shift of inactivation，prolonging fast recovery time from inactivation, and increasing entrance into the intermediate inactivation. These results suggest the mutation R1629Q does not affect channel expression and its ability to generate inward current in HEK293 cells, possibly because the mutation does not grossly disrupt the cardiac sodium channel folding, trafficking or function. But, the abnormality in electrophysiological properties caused by R1629Q mutation significantly disturbs the sodium channel activity responsible for increased transmural of dispersion repolarization, and might subsequently lead to phase 2 reentrant extrasystoles, and predispose the patient to VT and VF [21,31].

Interestingly, many evidences revealed that the S4 segment was postulated to function as voltage sensors in voltage-gate channels. However, DI-III are primarily involved in activation, whereas DIV has a distinct role in inactivation by applying specific toxins [32], site-directed fluorescence labeling [33] and paddle chimeras methods [23]. Many studies suggested that mutations in the DIV voltage sensors may affect slow and fast inactivation of Na_v_1.5 and other Na_v_ channels, exhibiting a possible tight coupling between the DIV voltage sensors and inactivation [23-26,32].Recently, Chanda and coworkers [34] showed that activation of the DIV voltage sensor might induced a conformational change of the pore necessary for fast inactivation to occur. However, it remains unclear whether DIV voltage sensors involve in intermediate inactivation which plays an important role in determine the channels availability during firing action potential. Our findings further suggest that the S4 segments in DIV may affect intermediate inactivation gating process and its stability of sodium channels. Moreover, there are a growing number of reports that mutations associated with *SCN5A* channelopathies resided in the voltage-sensor domain [10].For example, *SCN5A* mutations preferentially inclined to occur in the S4 segment in DCM patients, and among positive charge clusters acted as voltage sensor [35,36]. These mutations resulted in the functional defects of sodium channels with synthesis of a channel protein altered gating kinetics rather than reduced the current density [10,37]. Since the DIV-S4 plays a critical role in the inactivation of sodium channels, it was hardly surprising that R1629Q mutation altered the availability and intermediate inactivation of Na_v_1.5 channels.

In conclusion, we identified a heterozygous human mutation (R1629Q) in the voltage sensor of DIV-S4, causing malignant phenotype of a lethal arrhythmia secondary to VT/VF in a Chinese Han family. Although the mutation, R1629Q, has no significant effects on current density or steady-state activation, it can disrupts the electrophysiological characteristics of Na_v_1.5 channel by hyperpolarizing shift in steady–state inactivation, decreasing the decay time course, preferentially enhancing intermediate inactivation and prolonging recovery time from inactivation. Our findings may facilitate the understanding of arrhythmogenesis mechanisms of BrS and the role of DIV-S4 in Na_v_1.5 channels.
